# 2-Hy­droxy-*N*′-(5-hy­droxy-2-nitro­benzyl­idene)-3-methyl­benzohydrazide

**DOI:** 10.1107/S1600536812002437

**Published:** 2012-02-04

**Authors:** Zhao-Fu Zhu, Li-Juen Shao, Xi-Hai Shen

**Affiliations:** a Department of Chemistry, Hebei Normal University of Science and Technology, Qinhuangdao 066600, People’s Republic of China

## Abstract

The title compound, C_15_H_13_N_3_O_5_, was prepared by condensing 5-hy­droxy-2-nitro­benzaldehyde and 2-hy­droxy-3-methyl­benzohydrazide in methanol. The two benzene rings make a dihedral angle of 3.9 (3)°. An intra­molecular O—H⋯O hydrogen bond is observed. The crystal structure is stabilized by inter­molecular O—H⋯O and N—H⋯O hydrogen bonds, and C—H⋯O and π–π inter­actions [centroid–centroid distances = 3.5658 (17)–3.9287 (19) Å].

## Related literature

For the crystal structures of similar hydrazone compounds, see: Fun *et al.* (2011 ▶); Horkaew *et al.* (2011 ▶); Zhi *et al.* (2011 ▶); Huang & Wu (2010 ▶); Shen *et al.* (2012 ▶); Zhu *et al.* (2012 ▶).
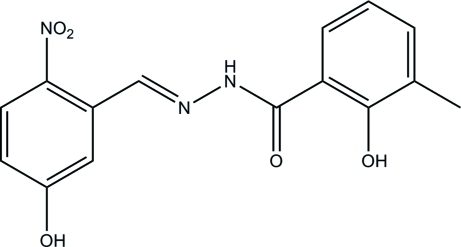


## Experimental

### 

#### Crystal data

C_15_H_13_N_3_O_5_
*M* *_r_* = 315.28
Triclinic, 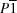

*a* = 7.643 (2) Å
*b* = 9.055 (3) Å
*c* = 10.876 (3) Å
α = 84.865 (2)°
β = 72.732 (2)°
γ = 77.479 (2)°
*V* = 701.4 (4) Å^3^
*Z* = 2
Mo *K*α radiation
μ = 0.12 mm^−1^
*T* = 298 K
0.18 × 0.17 × 0.13 mm

#### Data collection

Bruker SMART CCD area-detector diffractometer
Absorption correction: multi-scan (*SADABS*; Bruker, 2001 ▶) *T*_min_ = 0.980, *T*_max_ = 0.985
4694 measured reflections
2942 independent reflections
1788 reflections with *I* > 2σ(*I*)
*R*_int_ = 0.025

#### Refinement

*R*[*F* ^2^ > 2σ(*F* ^2^)] = 0.051
*wR*(*F* ^2^) = 0.132
*S* = 1.01
2942 reflections
214 parameters
1 restraint
H atoms treated by a mixture of independent and constrained refinement
Δρ_max_ = 0.21 e Å^−3^
Δρ_min_ = −0.22 e Å^−3^

### 

Data collection: *SMART* (Bruker, 2007 ▶); cell refinement: *SAINT* (Bruker, 2007 ▶); data reduction: *SAINT*; program(s) used to solve structure: *SHELXS97* (Sheldrick, 2008 ▶); program(s) used to refine structure: *SHELXL97* (Sheldrick, 2008 ▶); molecular graphics: *SHELXTL* (Sheldrick, 2008 ▶); software used to prepare material for publication: *SHELXTL*.

## Supplementary Material

Crystal structure: contains datablock(s) global, I. DOI: 10.1107/S1600536812002437/su2366sup1.cif

Structure factors: contains datablock(s) I. DOI: 10.1107/S1600536812002437/su2366Isup2.hkl

Supplementary material file. DOI: 10.1107/S1600536812002437/su2366Isup3.cml

Additional supplementary materials:  crystallographic information; 3D view; checkCIF report

## Figures and Tables

**Table 1:** Hydrogen-bond geometry (Å, °)

*D*—H⋯*A*	*D*—H	H⋯*A*	*D*⋯*A*	*D*—H⋯*A*
O5—H5⋯O4	0.82	1.83	2.552 (2)	146
O3—H3⋯O4^i^	0.82	1.97	2.775 (2)	168
N3—H3*B*⋯O2^ii^	0.89 (2)	2.53 (2)	3.397 (3)	167 (2)
C6—H6⋯O3^i^	0.93	2.54	3.306 (3)	140
C7—H7⋯O1^ii^	0.93	2.59	3.464 (3)	157
C14—H14⋯O2^ii^	0.93	2.44	3.325 (3)	159
C15—H15*C*⋯O2^iii^	0.96	2.54	3.488 (3)	170

Symmetry codes: (i) 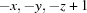; (ii) 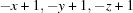; (iii) 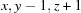.

**Fig. 1.**
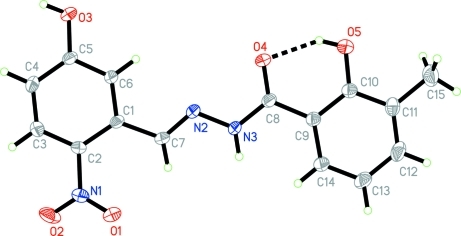
The molecular structure of the title molecule, with the atom numbering and displacement ellipsoids drawn at the 30% probability level. The intramolecular O—H···O hydrogen bond is drawn as a dashed line - see Table 1 for details.

**Fig. 2.**
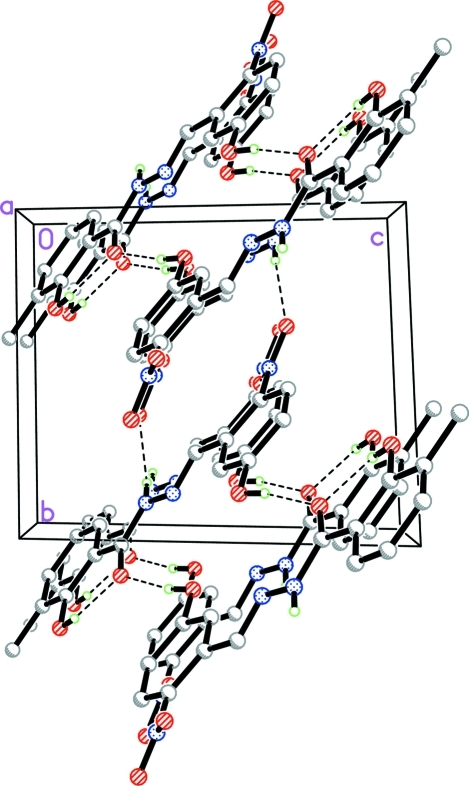
The crystal packing of the title compound, viewed along the a axis. Hydrogen bonds are drawn as dashed lines - see Table 1 for details.

## supplementary crystallographic information

### Comment

In the last few years, the synthesis and crystal structures of a number of hydrazone compounds have been reported (Fun *et al.*, 2011; Horkaew *et al.*, 2011; Zhi *et al.*, 2011; Huang & Wu, 2010). The compounds derived from 2-hydroxy-3-methylbenzohydrazide have seldom been reported As an extension of our work on such compounds (Shen *et al.*, 2012; Zhu *et al.*, 2012), we report herein on the crystal structure of the title compound.

In the molecule of the title compound there is an intramolecular O5—H5···O4 hydrogen bond (Table 1 and Fig. 1). The (C1—C6) and (C9—C14) benzene rings make a dihedral angle of 3.9 (3)°. All the bond values are within normal ranges and are comparable with those in similar compounds reported on by (Fun *et al.*, 2011; Horkaew *et al.*, 2011; Zhi *et al.*, 2011; Huang & Wu, 2010; Shen *et al.*, 2012; Zhu *et al.*, 2012).

In the crystal molecules are linked by intermolecular O—H···O and N—H···O hydrogen bonds and C—H···O interactions (Table 1 and Fig. 2). Moreover, there are also π–π interactions present involving molecules related by inversion centers [*Cg*1—*Cg*1^i^ 3.7989 (17) Å; *Cg*1—*Cg*2^ii^ 3.5658 (17) Å; *Cg*2—*Cg*2^iii^ 3.9287 (19) Å; symmetry codes: (i) -*x*, -*y* + 1, -*z* + 1; (ii) -*x* + 1, -*y*, -*z* + 1; (iii) -*x* + 1, -*y*, -*z* + 2; where *Cg*1 and *Cg*2 are the centroids of the (C1—C6) and (C9—C14) benzene rings, respectively].

### Experimental

5-Hydroxy-2-nitrobenzaldehyde (167.1 mg, 1.0 mmol) and 2-hydroxy-3-methylbenzohydrazide (166.2 mg, 1.0 mmol) were mixed in methanol (60 ml). The mixture was refluxed for 30 min, then cooled to room temperature, yielding a colourless solution. Colourless block-like crystals of the title compound were formed when the solution was evaporated in air for several days.

### Refinement

The amino H atom was located in a difference Fourier map and was refined isotropically, with the N—H distance restrained to 0.90 (1) Å. The OH and C-bound H-atoms were included in calculated positions and treated as riding atoms: O—H = 0.82 Å, C—H = 0.93, 0.97 and 0.96 Å for CH, CH_2_ and CH_3_, respectively, with *U*_iso_(H) = k × *U*_eq_(O,*C*), where k = 1.5 for OH and CH_3_ H-atoms, and k = 1.2 for all other H-atoms.

### Crystal data

**Table:**

C_15_H_13_N_3_O_5_	*Z* = 2
*M**_r_* = 315.28	*F*(000) = 328
Triclinic, *P*1	*D*_x_ = 1.493 Mg m^−^^3^
Hall symbol: -P 1	Mo *K*α radiation, λ = 0.71073 Å
*a* = 7.643 (2) Å	Cell parameters from 907 reflections
*b* = 9.055 (3) Å	θ = 2.3–26.3°
*c* = 10.876 (3) Å	µ = 0.12 mm^−^^1^
α = 84.865 (2)°	*T* = 298 K
β = 72.732 (2)°	Block, colourless
γ = 77.479 (2)°	0.18 × 0.17 × 0.13 mm
*V* = 701.4 (4) Å^3^	

### Data collection

**Table:**

Bruker SMART CCD area-detector diffractometer	2942 independent reflections
Radiation source: fine-focus sealed tube	1788 reflections with *I* > 2σ(*I*)
Graphite monochromator	*R*_int_ = 0.025
ω scans	θ_max_ = 27.0°, θ_min_ = 2.3°
Absorption correction: multi-scan (*SADABS*; Bruker, 2001)	*h* = −9→9
*T*_min_ = 0.980, *T*_max_ = 0.985	*k* = −11→10
4694 measured reflections	*l* = −13→10

### Refinement

**Table:**

Refinement on *F*^2^	Primary atom site location: structure-invariant direct methods
Least-squares matrix: full	Secondary atom site location: difference Fourier map
*R*[*F*^2^ > 2σ(*F*^2^)] = 0.051	Hydrogen site location: inferred from neighbouring sites
*wR*(*F*^2^) = 0.132	H atoms treated by a mixture of independent and constrained refinement
*S* = 1.01	*w* = 1/[σ^2^(*F*_o_^2^) + (0.0593*P*)^2^] where *P* = (*F*_o_^2^ + 2*F*_c_^2^)/3
2942 reflections	(Δ/σ)_max_ < 0.001
214 parameters	Δρ_max_ = 0.21 e Å^−^^3^
1 restraint	Δρ_min_ = −0.22 e Å^−^^3^

### Special details

**Table:**

Geometry. All e.s.d.'s (except the e.s.d. in the dihedral angle between two l.s. planes) are estimated using the full covariance matrix. The cell e.s.d.'s are taken into account individually in the estimation of e.s.d.'s in distances, angles and torsion angles; correlations between e.s.d.'s in cell parameters are only used when they are defined by crystal symmetry. An approximate (isotropic) treatment of cell e.s.d.'s is used for estimating e.s.d.'s involving l.s. planes.
Refinement. Refinement of *F*^2^ against ALL reflections. The weighted *R*-factor *wR* and goodness of fit *S* are based on *F*^2^, conventional *R*-factors *R* are based on *F*, with *F* set to zero for negative *F*^2^. The threshold expression of *F*^2^ > σ(*F*^2^) is used only for calculating *R*-factors(gt) *etc*. and is not relevant to the choice of reflections for refinement. *R*-factors based on *F*^2^ are statistically about twice as large as those based on *F*, and *R*- factors based on ALL data will be even larger.

### Fractional atomic coordinates and isotropic or equivalent isotropic displacement parameters (Å^2^)

**Table:**

	*x*	*y*	*z*	*U*_iso_*/*U*_eq_	
N1	0.3407 (2)	0.5111 (2)	0.34762 (17)	0.0392 (4)	
N2	0.3357 (2)	0.12755 (18)	0.60779 (16)	0.0378 (4)	
N3	0.4581 (2)	0.09322 (19)	0.68114 (17)	0.0387 (5)	
O1	0.4907 (2)	0.46271 (18)	0.36935 (16)	0.0566 (5)	
O2	0.3020 (2)	0.63840 (17)	0.30078 (17)	0.0619 (5)	
O3	−0.1905 (2)	0.15022 (17)	0.43917 (16)	0.0513 (5)	
H3	−0.2328	0.1533	0.3778	0.077*	
O4	0.35835 (19)	−0.12443 (16)	0.74968 (15)	0.0485 (4)	
O5	0.4783 (2)	−0.29790 (17)	0.91796 (16)	0.0548 (5)	
H5	0.4052	−0.2619	0.8761	0.082*	
C1	0.2028 (2)	0.2941 (2)	0.46606 (19)	0.0313 (5)	
C2	0.2020 (2)	0.4156 (2)	0.37541 (19)	0.0317 (5)	
C3	0.0716 (3)	0.4480 (2)	0.3073 (2)	0.0377 (5)	
H3A	0.0729	0.5299	0.2492	0.045*	
C4	−0.0602 (3)	0.3606 (2)	0.3242 (2)	0.0390 (5)	
H4	−0.1462	0.3816	0.2768	0.047*	
C5	−0.0633 (3)	0.2405 (2)	0.4132 (2)	0.0351 (5)	
C6	0.0654 (3)	0.2105 (2)	0.4831 (2)	0.0343 (5)	
H6	0.0594	0.1312	0.5438	0.041*	
C7	0.3329 (3)	0.2528 (2)	0.5452 (2)	0.0375 (5)	
H7	0.4112	0.3167	0.5493	0.045*	
C8	0.4651 (3)	−0.0391 (2)	0.7500 (2)	0.0352 (5)	
C9	0.6023 (3)	−0.0770 (2)	0.8241 (2)	0.0349 (5)	
C10	0.6006 (3)	−0.2070 (2)	0.9055 (2)	0.0371 (5)	
C11	0.7307 (3)	−0.2501 (2)	0.9763 (2)	0.0427 (5)	
C12	0.8620 (3)	−0.1626 (3)	0.9628 (2)	0.0536 (7)	
H12	0.9497	−0.1900	1.0086	0.064*	
C13	0.8668 (3)	−0.0341 (3)	0.8822 (3)	0.0577 (7)	
H13	0.9573	0.0230	0.8743	0.069*	
C14	0.7382 (3)	0.0073 (2)	0.8151 (2)	0.0470 (6)	
H14	0.7413	0.0937	0.7620	0.056*	
C15	0.7271 (4)	−0.3903 (3)	1.0606 (2)	0.0660 (8)	
H15A	0.8153	−0.3978	1.1091	0.099*	
H15B	0.7598	−0.4775	1.0081	0.099*	
H15C	0.6039	−0.3856	1.1187	0.099*	
H3B	0.538 (3)	0.152 (2)	0.679 (2)	0.080*	

### Atomic displacement parameters (Å^2^)

**Table:**

	*U*^11^	*U*^22^	*U*^33^	*U*^12^	*U*^13^	*U*^23^
N1	0.0418 (10)	0.0344 (10)	0.0408 (11)	−0.0144 (8)	−0.0062 (9)	−0.0007 (8)
N2	0.0381 (9)	0.0390 (10)	0.0454 (12)	−0.0133 (8)	−0.0227 (9)	0.0031 (9)
N3	0.0425 (10)	0.0379 (10)	0.0477 (12)	−0.0160 (8)	−0.0279 (9)	0.0080 (9)
O1	0.0478 (9)	0.0662 (11)	0.0698 (13)	−0.0300 (8)	−0.0291 (9)	0.0142 (9)
O2	0.0599 (10)	0.0369 (9)	0.0838 (14)	−0.0183 (8)	−0.0116 (9)	0.0158 (9)
O3	0.0527 (9)	0.0545 (10)	0.0658 (12)	−0.0282 (8)	−0.0365 (8)	0.0104 (8)
O4	0.0516 (9)	0.0462 (9)	0.0647 (11)	−0.0263 (7)	−0.0348 (8)	0.0129 (8)
O5	0.0548 (10)	0.0532 (10)	0.0677 (13)	−0.0277 (8)	−0.0289 (9)	0.0199 (8)
C1	0.0320 (10)	0.0303 (11)	0.0356 (12)	−0.0080 (8)	−0.0134 (9)	−0.0050 (9)
C2	0.0308 (10)	0.0282 (10)	0.0359 (12)	−0.0093 (8)	−0.0067 (9)	−0.0015 (9)
C3	0.0411 (12)	0.0344 (12)	0.0371 (13)	−0.0061 (9)	−0.0130 (10)	0.0034 (9)
C4	0.0375 (11)	0.0436 (13)	0.0406 (14)	−0.0045 (10)	−0.0208 (10)	−0.0004 (10)
C5	0.0333 (10)	0.0344 (11)	0.0430 (14)	−0.0104 (9)	−0.0156 (10)	−0.0034 (10)
C6	0.0383 (11)	0.0307 (11)	0.0404 (13)	−0.0113 (9)	−0.0192 (10)	0.0031 (9)
C7	0.0386 (11)	0.0365 (12)	0.0460 (14)	−0.0168 (9)	−0.0191 (10)	0.0017 (10)
C8	0.0330 (11)	0.0368 (12)	0.0400 (13)	−0.0095 (9)	−0.0144 (10)	−0.0023 (10)
C9	0.0374 (11)	0.0325 (11)	0.0377 (13)	−0.0076 (9)	−0.0141 (10)	−0.0031 (9)
C10	0.0384 (11)	0.0371 (12)	0.0369 (13)	−0.0100 (9)	−0.0104 (10)	−0.0028 (10)
C11	0.0476 (13)	0.0452 (13)	0.0346 (13)	−0.0012 (10)	−0.0161 (10)	−0.0030 (10)
C12	0.0589 (14)	0.0553 (15)	0.0584 (17)	−0.0034 (12)	−0.0385 (13)	−0.0071 (13)
C13	0.0598 (15)	0.0497 (15)	0.083 (2)	−0.0193 (12)	−0.0432 (14)	−0.0026 (14)
C14	0.0536 (13)	0.0389 (13)	0.0619 (17)	−0.0181 (11)	−0.0322 (12)	0.0045 (11)
C15	0.0682 (16)	0.0716 (18)	0.0578 (18)	−0.0093 (14)	−0.0270 (14)	0.0207 (14)

### Geometric parameters (Å, º)

**Table:**

N1—O1	1.219 (2)	C4—H4	0.9300
N1—O2	1.230 (2)	C5—C6	1.382 (3)
N1—C2	1.457 (2)	C6—H6	0.9300
N2—C7	1.269 (2)	C7—H7	0.9300
N2—N3	1.372 (2)	C8—C9	1.471 (3)
N3—C8	1.354 (3)	C9—C14	1.393 (3)
N3—H3B	0.889 (10)	C9—C10	1.408 (3)
O3—C5	1.354 (2)	C10—C11	1.402 (3)
O3—H3	0.8200	C11—C12	1.376 (3)
O4—C8	1.240 (2)	C11—C15	1.499 (3)
O5—C10	1.344 (2)	C12—C13	1.392 (3)
O5—H5	0.8200	C12—H12	0.9300
C1—C6	1.384 (2)	C13—C14	1.363 (3)
C1—C2	1.410 (3)	C13—H13	0.9300
C1—C7	1.470 (3)	C14—H14	0.9300
C2—C3	1.379 (3)	C15—H15A	0.9600
C3—C4	1.372 (3)	C15—H15B	0.9600
C3—H3A	0.9300	C15—H15C	0.9600
C4—C5	1.389 (3)		
			
O1—N1—O2	122.22 (18)	C1—C7—H7	120.9
O1—N1—C2	119.73 (17)	O4—C8—N3	120.40 (18)
O2—N1—C2	118.04 (18)	O4—C8—C9	121.74 (19)
C7—N2—N3	116.56 (16)	N3—C8—C9	117.87 (17)
C8—N3—N2	118.53 (16)	C14—C9—C10	118.08 (19)
C8—N3—H3B	120.2 (16)	C14—C9—C8	123.11 (19)
N2—N3—H3B	121.0 (16)	C10—C9—C8	118.78 (18)
C5—O3—H3	109.5	O5—C10—C11	116.37 (19)
C10—O5—H5	109.5	O5—C10—C9	122.50 (18)
C6—C1—C2	116.38 (17)	C11—C10—C9	121.12 (19)
C6—C1—C7	118.11 (18)	C12—C11—C10	118.0 (2)
C2—C1—C7	125.49 (17)	C12—C11—C15	122.0 (2)
C3—C2—C1	121.46 (17)	C10—C11—C15	119.9 (2)
C3—C2—N1	116.86 (17)	C11—C12—C13	121.7 (2)
C1—C2—N1	121.67 (17)	C11—C12—H12	119.1
C4—C3—C2	120.70 (19)	C13—C12—H12	119.1
C4—C3—H3A	119.6	C14—C13—C12	119.6 (2)
C2—C3—H3A	119.6	C14—C13—H13	120.2
C3—C4—C5	119.12 (19)	C12—C13—H13	120.2
C3—C4—H4	120.4	C13—C14—C9	121.4 (2)
C5—C4—H4	120.4	C13—C14—H14	119.3
O3—C5—C6	116.53 (18)	C9—C14—H14	119.3
O3—C5—C4	123.56 (18)	C11—C15—H15A	109.5
C6—C5—C4	119.89 (18)	C11—C15—H15B	109.5
C5—C6—C1	122.41 (18)	H15A—C15—H15B	109.5
C5—C6—H6	118.8	C11—C15—H15C	109.5
C1—C6—H6	118.8	H15A—C15—H15C	109.5
N2—C7—C1	118.21 (18)	H15B—C15—H15C	109.5
N2—C7—H7	120.9		

### Hydrogen-bond geometry (Å, º)

**Table:**

*D*—H···*A*	*D*—H	H···*A*	*D*···*A*	*D*—H···*A*
O5—H5···O4	0.82	1.83	2.552 (2)	146
O3—H3···O4^i^	0.82	1.97	2.775 (2)	168
N3—H3*B*···O2^ii^	0.89 (2)	2.53 (2)	3.397 (3)	167 (2)
C6—H6···O3^i^	0.93	2.54	3.306 (3)	140
C7—H7···O1^ii^	0.93	2.59	3.464 (3)	157
C14—H14···O2^ii^	0.93	2.44	3.325 (3)	159
C15—H15*C*···O2^iii^	0.96	2.54	3.488 (3)	170

Symmetry codes: (i) −*x*, −*y*, −*z*+1; (ii) −*x*+1, −*y*+1, −*z*+1; (iii) *x*, *y*−1, *z*+1.
